# IgG Fc-binding protein positively regulates the assembly of pore-forming protein complex βγ-CAT evolved to drive cell vesicular delivery and transport

**DOI:** 10.1016/j.jbc.2023.104717

**Published:** 2023-04-15

**Authors:** Xianling Bian, Ziru Si, Qiquan Wang, Lingzhen Liu, Zhihong Shi, Changlin Tian, Wenhui Lee, Yun Zhang

**Affiliations:** 1 Hefei National Laboratory for Physical Sciences at Microscale, School of Life Sciences, Division of Life Sciences and Medicine, University of Science and Technology of China, Hefei, Anhui, China; 2 Key Laboratory of Animal Models and Human Disease Mechanisms of the Chinese Academy of Sciences/Engineering Laboratory of Peptides of the Chinese Academy of Sciences, Institute of Zoology, The Chinese Academy of Sciences, Kunming, Yunnan, China; 3 Human Aging Research Institute (HARI) and School of Life Sciences, Nanchang University, Nanchang, Jiangxi, China; 4 Center for Excellence in Animal Evolution and Genetics, Chinese Academy of Sciences, Kunming, Yunnan, China

**Keywords:** IgG Fc-binding protein, pore-forming protein, trefoil factor, cell delivery, endolysosomal systems

## Abstract

Cell membranes form barriers for molecule exchange between the cytosol and the extracellular environments. βγ–CAT, a complex of pore-forming protein BmALP1 (two βγ-crystallin domains with an aerolysin pore-forming domain) and the trefoil factor BmTFF3, has been identified in toad *Bombina maxima*. It plays pivotal roles, *via* inducing channel formation in various intracellular or extracellular vesicles, as well as in nutrient acquisition, maintaining water balance, and antigen presentation. Thus, such a protein machine should be tightly regulated. Indeed, BmALP3 (a paralog of BmALP1) oxidizes BmALP1 to form a water-soluble polymer, leading to dissociation of the βγ–CAT complex and loss of biological activity. Here, we found that the *B. maxima* IgG Fc-binding protein (FCGBP), a well-conserved vertebrate mucin-like protein with unknown functions, acted as a positive regulator for βγ–CAT complex assembly. The interactions among FCGBP, BmALP1, and BmTFF3 were revealed by co-immunoprecipitation assays. Interestingly, FCGBP reversed the inhibitory effect of BmALP3 on the βγ–CAT complex. Furthermore, FCGBP reduced BmALP1 polymers and facilitated the assembly of βγ-CAT with the biological pore-forming activity in the presence of BmTFF3. Our findings define the role of FCGBP in mediating the assembly of a pore-forming protein machine evolved to drive cell vesicular delivery and transport.

Cellular membranes are crucial for functional compartmentalization and the survival of cells. Membrane transporters including the solute carrier superfamily act in uptake of small substances such as cell nutrients glucose and amino acids (1, 2). Additionally, endolysosomal pathways are essential for cells to import diverse insoluble and macromolecular materials including proteins and fatty acid nutrients (3, 4). Pore-forming proteins (PFPs) in various families with structural folds are widely distributed in all kingdoms of life (5, 6, 7, 8, 9). PFPs have long been recognized as either pore-forming toxin for microbial infection (6, 10, 11) or host immune executors (12, 13, 14, 15). In particular, aerolysin is a bacterial β-barrel pore-forming toxin (16, 17). However, a diverse array of aerolysin family PFPs (af-PFPs; previously referred to as aerolysin-like proteins, ALPs) have been identified in various animals and plant species (16, 17, 18, 19, 20).

BmALP1, an af-PFP in toad *Bombina maxima*, interacts with trefoil factor (TFF) BmTFF3 to form a membrane-active PFP complex named βγ–CAT (21, 22, 23). This protein complex targets acidic glycosphingolipids in lipid rafts of cell plasma membranes *via* a double-receptor binding model (23). It also drives import and export of extracellular materials and/or plasma membrane components through endo-lysosomal systems (24, 25, 26). This PFP oligomerizes to form channels with a functional diameter approximately 1.5 to 2.0 nm on endolysosomes, facilitating material exchange between these intracellular organelles and the cytosol (14, 21, 26, 27). Thus, the βγ–CAT complex is a multifunctional PFP that plays diverse physiological roles in *B. maxima* depending on distinct cell contexts and environmental cues. Accordingly, the roles of βγ–CAT in immune defense were first documented (14, 27, 28, 29, 30).

In starved toad cells, βγ–CAT drives the production of protein nutrient-containing vesicles, called nutrisomes. These specific vesicles fuse with lysosomes to hydrolyze imported proteins and the resulting amino acid products are released to cytosol to support the nutrient supply and energy fuel in starved cells (31). Alternatively, specific intracellular vesicles induced by βγ–CAT, which contain imported extracellular substances, such as water with ions (26) and albumin-bound fatty acids (24), are exported out of cells by exosome release, resulting in intercellular delivery of the cargo molecules for water acquisition and tissue parenchymal cell nutrient supply, respectively. Thus, βγ–CAT defines a secretory (soluble) endolysosomal channel pathway, representing a novel PFP system evolved to mediate cell vesicular delivery and transport (24, 25, 26). Reasonably, the βγ–CAT pathway should be regulated tightly.

Interestingly, af-PFPs in toad *B. maxima* contain a conserved C-terminal cysteine that is a key regulatory site *via* redox reactions. Consequently, BmALP1 is oxidized into a homodimer and water-soluble high molecular weight polymer by BmALP3, a paralog of BmALP1, *via* disulfide bond exchange, which negatively regulates the assembly and biological functions of the βγ–CAT complex (32). Considering the important physiological roles of βγ–CAT, positive regulatory elements should exist in the toad to promote activation and assembly of βγ–CAT. Here, by screening potential βγ-CAT–associated proteins, we found that IgG Fc-binding protein (FCGBP), a mucin-like protein highly conserved in vertebrates with unknown functions (33, 34), interacted physically with βγ-CAT subunits in toad *B. maxima* skin secretions. Further experiments demonstrated that FCGBP possessed the capacity to reduce oxidized BmALP1 and served as a scaffold for the interaction of BmALP1 with BmTFF3 to form the βγ–CAT complex. Additionally, *B. maxima* peroxiredoxin 6 (Prdx6) and thioredoxin (Trx) restored and enhanced the effect of FCGBP on βγ-CAT assembly.

## Results

### FCGBP interacts with βγ-CAT subunits BmALP1 and BmTFF3

**Figure 1:**
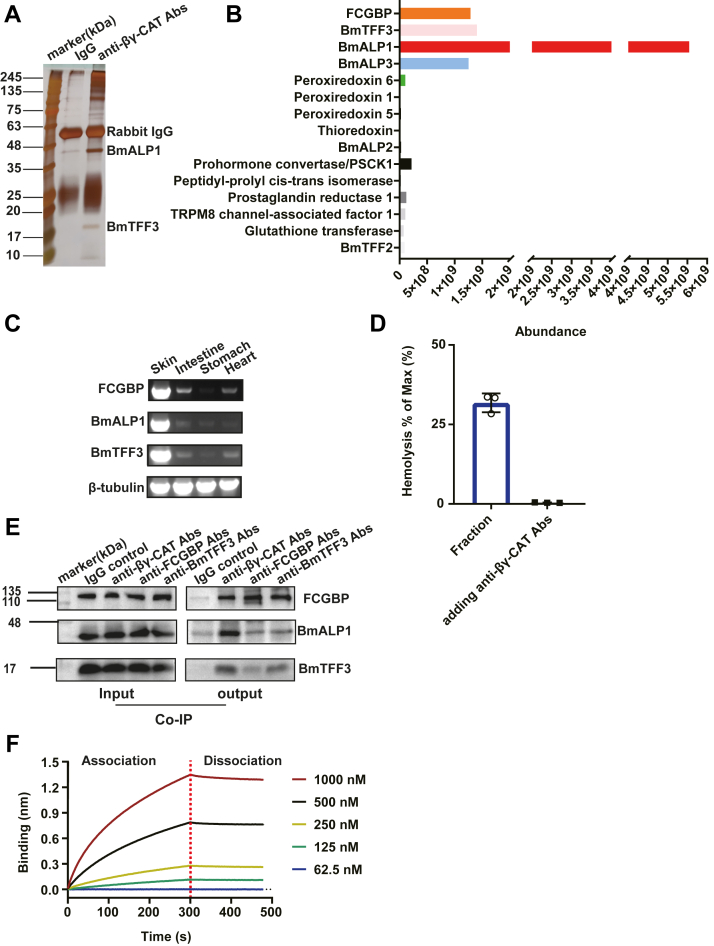
***Bombina maxima* FCGBP interacts with BmALP1 and BmTFF3 (subunits of βγ-CAT).***A*, the IP result using rabbit IgG and anti-βγ-CAT antibodies. *B*, identified proteins from IP results by LC-MS. *C*, expression profiles of FCGBP, BmALP1, and BmTFF3 mRNAs in tissues (skin, intestine, stomach, and heart). β-Tubulin was used as the control. *D*, hemolysis assays of the elution fraction from the pull-down assay of the Sepharose 4B-anti-FCGBP antibody column with *B. maxima* secretions as the input. *E*, Western blots showing the results of Co-IP assays using anti-FCGBP, anti-βγ-CAT, and anti-BmTFF3 antibodies. *B. maxima* secretions as the input were incubated with protein A slurry beads (*left*) and outputted after eluting using elution buffer (*right*). Rabbit IgG was used as the negative control. *F*, association and Dissociation data of FCGBP and βγ-CAT at serial concentrations were analyzed by the BLI method of a global fit and a 1:1 binding model, with *K*_D_ at 2.12 × 10^−8^ M, *K*_D_ error at 1.37 × 10^−9^ M, full X^2^ at 0.6163, and full R^2^ at 0.999. Ab, antibody; BLI, bio-layer interferometry; IP, immunoprecipitation; TFF, trefoil factor.

To identify proteins that interact with βγ-CAT, *B. maxima* skin secretions were subjected to protein A columns, respectively coupled with antibodies for immunoprecipitation (IP) assays. Some protein bands were found by comparing the products of the protein A column coupled with polyclonal anti-βγ-CAT antibodies with those of the protein A column coupled with rabbit IgG (Fig. 1*A*). These unknown proteins that indirectly or directly interacted with βγ-CAT were identified and listed as potential candidates with high abundance and oxidoreductases through an IP/LC-MS method (Fig. 1*B*). In particular, except for BmALP3 (a negative regulator of βγ-CAT) and the two BmALP1 and BmTFF3 subunits of βγ-CAT, FCGBP with high abundance and some oxidoreductases were identified such as Prdx6 and Trx (Fig. 1*B*). Transcriptomic analysis of the toad *B. maxima* skin (35) revealed a cDNA sequence encoding FCGBP. Semiquantitative PCR of FCGBP showed that the mRNA displayed a tissue distribution pattern and was abundant in skin and intestines. Reported FCGBP was widely expressed in mucosal surfaces and external secretions in humans and mice (36). It was also distributed in the secretory system. FCGBP was co-expressed with the BmALP1 and BmTFF3 subunits of βγ-CAT in various tissues, which was highly expressed and consistent with βγ-CAT in toad skin (Fig. 1*C*). Additionally, after pull-down assays using anti-FCGBP antibodies, the eluted solution induced hemolysis of human red blood cells (RBCs), which was also blocked by anti-βγ-CAT antibodies (Fig. 1*D*). This strongly suggested that FCGBP had a relationship with βγ-CAT. Furthermore, Co-IP assays using various antibodies, including anti-FCGBP, anti-CAT, and anti-BmTFF3, revealed significant interactions between FCGBP and βγ-CAT subunits (BmALP1 and BmTFF3) (Fig. 1*E*). The peptide fragment of BmALP1 was also determined (Fig. S1*A*). The bio-layer interferometry (BLI) method showed that FCGBP and βγ-CAT had a strong molecular interaction with a *K*_D_ value up to 2.12 × 10^−8^ M (Fig. 1*F*). These findings indicated an interaction between FCGBP and βγ-CAT subunits, suggesting that the newly identified FCGBP in toad skin secretions may be involved in the assembly and biological functions of βγ-CAT.

### Natural FCGBP in toad *B. maxima* skin secretions

**Figure 2:**
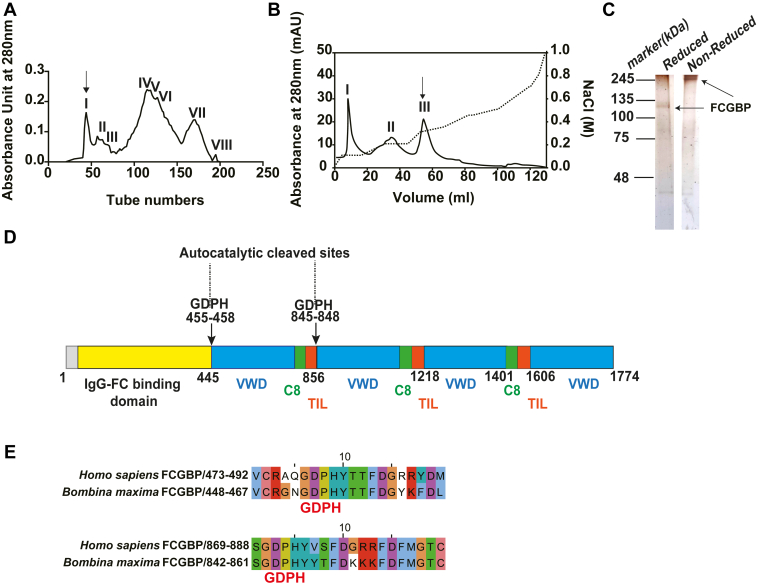
**Purification and identification of FCGBP from *Bombina maxima* secretions.***A*, isolation of FCGBP from *B. maxima* secretions by a Sephadex G-100 column. The *arrow* indicates FCGBP that was mainly found in peak I. *B*, resource Q anion-exchange column of peak I from gel filtration was found in peak III containing the target proteins (*arrow*). *C*, silver staining of the final product from antibody affinity chromatography of anti-FCGBP. SDS-PAGE was performed under reducing and nonreducing conditions. *D*, *B. maxima* skin FCGBP from transcriptome and proteosome analyses, its full-length amino acid sequence included vWD domains (*blue*), C8 cys-rich domains (*green*), TIL domains (*orange*), and an IgG-Fc-binding domain (*yellow*), which had two GDPH autocatalytic cleavage sites (*arrow*). *E*, sequence alignments of the autocatalytic cleavage sites of GDPH between FCGBP and human FCGBP. vWD, von Willebrand type D.

FCGBP was highly present in the skin of *B. maxima*. Thus, to better understand the molecular characteristics and functions of FCGBP, *B. maxima* skin secretions were separated on a Sephadex G-100 column. FCGBP was distributed in peaks I and II (Figs. 2*A* and S2*A*). The peak containing FCGBP was collected for isolation by an anion exchange column which produced three protein peaks (Fig. 2*B*). Peak III of anion exchanges containing FCGBP was injected into a Sepharose-4B-anti-FCGBP affinity column. The purified FCGBP had an apparent weight of 200 kDa in nonreduced SDS-PAGE. It could break different fragments in reduced SDS-PAGE and identified by MS (Figs. 2*C* and S2*B*). Data analysis of the *B. maxima* skin transcriptome and proteome indicated that FCGBP was 1774 amino acids with a theoretic molecular weight of 195.25 kDa. The domain architecture of FCGBP was similarly conserved to human FCGBP (Fig. S2*D*), which included an IgG Fc-binding domain at the N terminus and four repeated units that were composed of von Willebrand type D (vWD) and C8 cysteine-rich and trypsin inhibitor-like domains (Fig. 2*D*). Furthermore, sequence alignments revealed that the protein shared 35.4% sequence identity with human FCGBP, indicating conservation of FCGBPs in vertebrates (Fig. S2*D*). The FCGBP sequence was shared two auto-cleaved sites of GDPH at amino acids 445–448 and 845–848. (Fig. 2*E*), which was autocatalytically cleaved to generate different fragments and was connected by disulfide bonds as reported previously (34).

### FCGBP positively regulates formation of the βγ–CAT complex

A paralog of the BmALP1 subunit (named BmALP3) of βγ-CAT has been identified in *B. maxima*. These two β-PFPs (*i.e.*, BmALP1 and BmALP3) contain a conserved cysteine in their C-terminal regions, which is also highly conserved in vertebrate β-PFPs from fish to reptiles (32). The BmALP3 homodimer linked by disulfide bonds specifically oxidizes BmALP1 into its own homodimer *via* disulfide bond formation as well as water-soluble higher molecular weight polymers, which leads to disassociation of the βγ–CAT complex and loss of its biological activity. Additionally, the hemolytic activity is an indication of the presence of biologically active βγ–CAT complex, which is a best and fast method.

#### FCGBP restores the hemolytic activity of βγ-CAT

**Figure 3:**
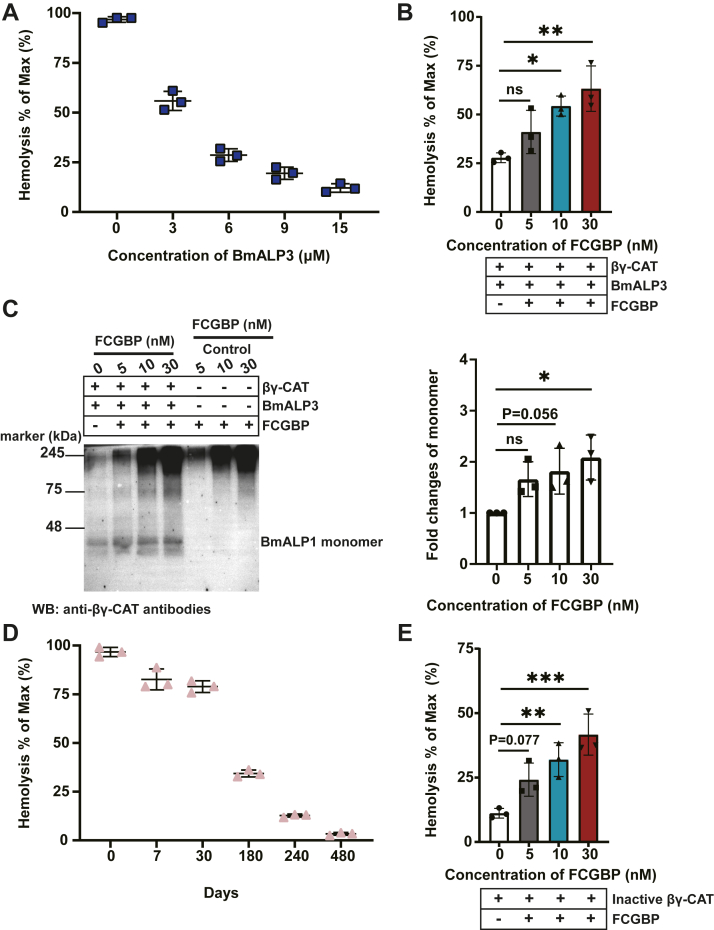
**FCGBP restores hemolytic activity of the βγ–CAT complex.***A*, βγ-CAT (5 nM) and various concentrations of BmALP3 were incubated with human red blood cells (RBCs) at 37 °C for 30 min and then hemolysis was assessed. *B*, various concentrations of FCGBP were incubated with BmALP3 (6 μM) and βγ-CAT (5 nM) and then incubated with RBCs for hemolysis assays. *C*, changes of BmALP1 monomers band in the presence of FCGBP were analyzed by Western blotting under nonreducing conditions (*left*). The change in the amount of BmALP1 monomers was semiquantified by ImageJ (*right*). *D*, hemolytic activity of βγ-CAT stored at 4 °C for various times was assessed by reacting with human RBCs at 37 °C. *E*, 240-day-stored βγ-CAT was incubated with fresh FCGBP at various concentrations and then incubated with RBCs for hemolysis assays. Data represent the mean ± SD of triplicate samples. ∗*p* < 0.05, ∗∗*p* < 0.01, ∗∗∗*p* < 0.001, and ∗∗∗∗*p* < 0.0001 by Ordinary one-way ANOVA test. ALP, aerolysin-like protein.

In a preliminary experiment, BmALP3 did inhibit βγ-CAT activity in a dose-dependent manner (Fig. 3*A*). Interestingly, the negative regulatory action of BmALP3 (6 μM) on hemolytic activity of βγ-CAT was dose-dependently restored by FCGBPs, whereas a single FCGBP had no hemolytic activity (Figs. 3*B* and S3*A*). In the presence of 10 and 30 nM FCGBP, the hemolytic activity was recovered by 27% and 36%, respectively (Fig. 3*B*). Conversely, rePrdx6 or reTrx alone instead of FCGBP did not restore βγ-CAT hemolytic activity, even at a dose up to approximately 3.1 μM (Fig. S3*B*). Western blotting was used to detect the generation of BmALP1 monomers. In the presence of FCGBP, BmALP1 monomer bands were significantly observed (Fig. 3*C*). These data suggested that FCGBP had the ability to reverse the inhibition of βγ-CAT by BmALP3 and restore hemolytic activity of the complex.

Purified βγ–CAT complex underwent denaturation when stored alone at 4 °C, resulting in a dramatic loss of its biological activity. When purified βγ-CAT was stored alone for 240 days at 4 °C, hemolytic activity was strongly decreased to approximately 13% (Fig. 3*D*). Intriguingly, when various concentrations of fresh FCGBP were incubated with 240-day-stored βγ-CAT, the hemolytic activity of βγ-CAT was augmented in a dose-dependent manner (Fig. 3*E*). However, rePrdx6 or reTrx alone instead of FCGBP had no effect on βγ-CAT hemolytic activity (Fig. S3*C*). These findings revealed that FCGBP reversed the negative effect of BmALP3 on active βγ-CAT and restored the hemolytic activity of oxidized βγ-CAT.

#### FCGBP facilitates βγ-CAT formation in the presence of BmTFF3

**Figure 4:**
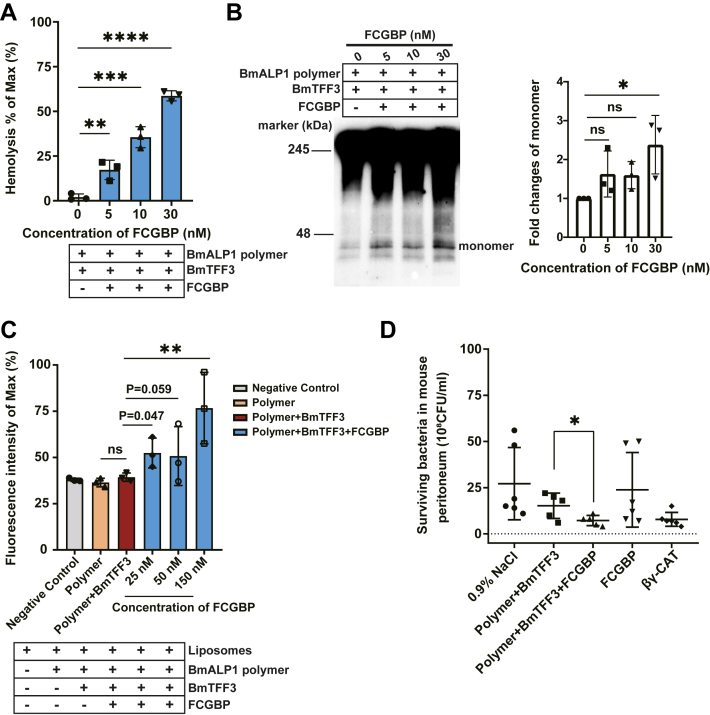
**FCGBP assembles the active βγ–CAT complex.***A*, hemolysis assays in which polymer (20 nM) and BmTFF3 (300 nM) were added to RBCs in the presence of FCGBP at various concentrations. *B*, changes of BmALP1 monomer bands were identified under nonreducing conditions in the presence of various concentrations of FCGBP by Western blotting. *Left*: Western blot results; *Right*: semiquantified amount of BmALP1 monomers bands were analyzed by ImageJ. *C*, dye release at which samples reacted for 2 min with liposomes encapsulating calcein was analyzed by the fluorescence intensity at 518 nm. All data significance represents the mean ± SD of triplicate samples. ∗*p* < 0.05, ∗∗*p* < 0.01, ∗∗∗*p* < 0.001, and ∗∗∗∗*p* < 0.0001 by Ordinary one-way ANOVA test. *D*, the number of peritoneal bacteria were counted in mice injected peritoneally with *Escherichia. coli* (ATCC 25922). See details in Experiment Procedures. At least five mice were included in each group. ∗*p* < 0.05, ∗∗*p* < 0.01, ∗∗∗*p* < 0.001, and ∗∗∗∗*p* < 0.0001 by unpaired *t* test. ALP, aerolysin-like protein; RBC, red blood cell.

During collection and isolation processes, because of the presence of BmALP3, a fraction of BmALP1 had been oxidized to BmALP1 polymers, which appeared in collected samples of toad *B. maxima* skin secretions. Thus, natural BmALP1 polymers were isolated by a series of purification procedures including gel filtration, anion exchanges, and affinity chromatography (Fig. S4, *A*–*C*). In hemolytic assays, isolated natural polymers at approximately 21 μg/ml did not display hemolytic activity and BmTFF3 itself and the mixture of natural polymers with BmTFF3 also did not possess any hemolytic activity (Fig. S4, *D* and *E*). However, interestingly, in the presence of BmTFF3, significantly increased hemolytic activity appeared in a dose-dependent manner after addition of various concentrations of FCGBP (Fig. 4*A*), which was also blocked by anti-βγ-CAT antibodies (Fig. S4*E*). This result indicated the appearance of the βγ–CAT complex. Moreover, BmALP1 monomer bands were significantly observed in Western blots (Fig. 4*B*). Because βγ-CAT has traditional pore-forming activity on cell membranes, various concentrations of FCGBP, BmTFF3, and BmALP1 polymers were premixed and added to 0.1 μm liposomes encapsulating 120 mM calcein which selfquenched at high concentrations and fluoresced at low concentrations, leading to dye release. The mean fluorescence intensity of maximum was measured up to 76.8% at 518 nm emission when FCGBP was added at 150 nM (Fig. 4*C*), suggesting generation of active βγ-CAT. Active βγ-CAT protects the host against microbial infection (30). Thus, we assessed *in vivo* FCGBP activity in mice. Bacterial counting in mice peritoneal fluids showed that mice injected FCGBP, polymers, and BmTFF3 had significant clearance on *Escherichia coli* and active βγ-CAT–injected mice as the positive control (Fig. 4*D*), suggesting that FCGBP assembled active βγ-CAT to stimulate mice anti-bacterial immune reactions *in vivo*. These findings implied that FCGBP reduced polymers to generate active βγ-CAT.

We also assessed the possible functions of Prdx6 and Trx in BmALP1 polymers reduction and complex formation under the same conditions as the above assays for FCGBP. Different from the positive effects of FCGBP, in the presence of recombinant Prdx6 or Trx at concentrations used up to 3.1 μM, neither reduction of BmALP1 polymer nor βγ-CAT hemolytic activity was detected (Fig. S4*F*), indicating that these oxidoreductases did not directly reduce oxidized BmALP1.

#### FCGBP assembles recombinant BmALP1 and BmTFF3

To further determine whether FCGBP facilitated generation of the βγ–CAT complex, reBmALP1 was expressed through the secreted expression system of *Pichia pastoris.* ReBmALP1 had polymeric forms of approximately 240 kDa (Fig. S5*A*). Hemolysis assays indicated that reBmALP1 polymers and BmTFF3 had no hemolytic activity, whereas addition of 30 nM FCGBP increased hemolysis significantly which was blocked by anti-βγ-CAT antibodies with rabbit IgG as the control. In the presence of FCGBP at serial concentrations, hemolytic activity of the mixture of FCGBP and reBmALP1 polymers with BmTFF3 on RBCs was significantly increased in a dose-dependent manner (Fig. S5*B*). And BmALP1 monomer bands were also significantly observed in Western blots (Fig. S5*C*). These results implied that FCGBP assembled reBmALP1 polymers and BmTFF3 to generate the βγ–CAT complex.

Collectively, these data showed that FCGBP, but not Prdx6 or Trx, was a positive regulator of βγ–CAT complex formation and its biological functions in toad *B. maxima*.

### Oxidoreductases Prdx6 and Trx restore FCGBP activity

**Figure 5:**
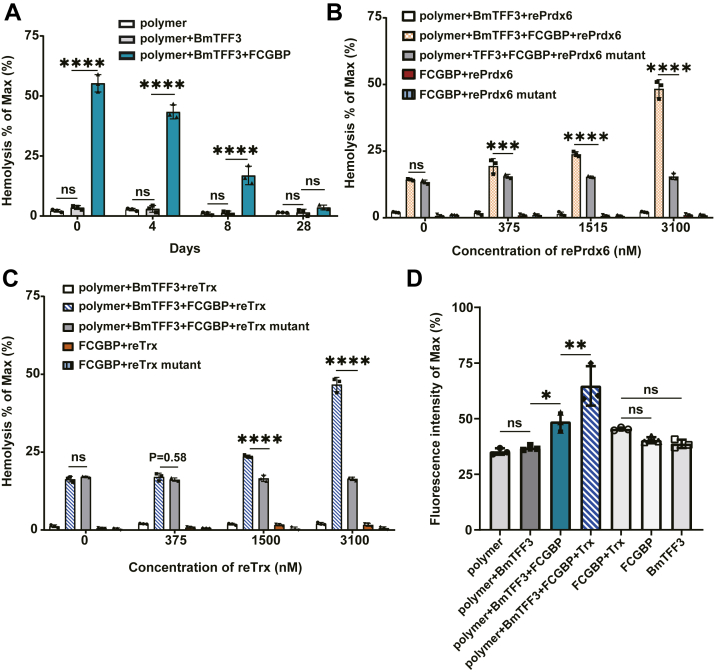
**Prdx6 or Trx restores the function FCGBP in βγ-CAT formation.***A*, functional activity of FCGBP stored at 4 °C for various time was assessed by hemolysis assays. FCGBP mixed with natural BmALP1 polymers and BmTFF3 was reacted with RBCs. *B*, functional activity of rePrdx6 was assessed by hemolysis with the mutant as the control. rePrdx6 mixed with FCGBP, polymers, and BmTFF3 was reacted with RBCs to determine whether hemolytic activity of βγ-CAT had increased. See details in Hemolysis assays of Experimental procedures. *C*, functional activity of reTrx was assessed by hemolysis with the mutant as the control. reTrx mixed with FCGBP, polymers, and BmTFF3 was reacted with RBCs to determine whether hemolytic activity of βγ-CAT had increased. See details in Hemolysis assays of Experimental procedures. *D*, dye release of samples reacted for 2 min with liposomes encapsulating calcein was indicated by the fluorescence intensity at 518 nm. Data represents the mean ± SD of triplicate samples. ∗*p* < 0.05, ∗∗*p* < 0.01, ∗∗∗*p* < 0.001, and ∗∗∗∗*p* < 0.0001 by Ordinary ANOVA test. ALP, aerolysin-like protein; RBC, red blood cell; TFF, trefoil factor.

Purified FCGBP stored alone at 4 °C underwent denaturation with dramatic loss of its biological activity in 7 days (Fig. 5*A*). When FCGBP was stored for 28 days, its effect on BmALP1 polymers was decreased strongly to approximately 1% (Fig. 5*A*). This suggested that FCGBP was composed of easily oxidized-free cystines and the status of oxidized and reduced free cystines might be changed by certain oxidoreductases. Interestingly, the protein sequence identities of reducing enzymes Prdx6 and Trx were highly conserved between toad *B. maxima* (35) and humans (86% and 70% sequence identities, respectively). Thus, experiments were carried out to determine whether Prdx6 and Trx restored the biological activity of naturally occurring FCGBP. Interestingly, Various concentrations of reprdx6 were reacted with a mixture of inactive FCGBP, natural purified polymers, and BmTFF3 to assess hemolysis of RBCs with reprdx6^(C46A, C90A)^ mutant as the control. rePrdx6 (3.1 μM) significantly restored the biological activity of FCGBP with being stored for 8 days (Fig. 5*B*). Similarly, reTrx (3.1 μM) also restored the biological activities of FCGBP (Fig. 5*C*). In dye release assays of pore formation in liposomes, FCGBP had no activity on liposomes. However, the fluorescence intensity was significantly increased after addition of 150 nM FCGBP. When FCGBP was reduced by Trx, significantly more dye was released than after addition of FCGBP stored for 8 days, resulting in a difference of up to 12.84% (Fig. 5*D*). These results suggested that the biological activity of FCGBP was restored by oxidoreductases Prdx6 and Trx *via* a redox reaction. These findings indicated FCGBP assembled active βγ-CAT and FCGBP with deceased activity reduced prdx6 and Trx.

## Discussion

Cell membranes with their selective permeability form barriers for molecule exchange between the cytosol and the extracellular environment (37, 38). Toad *B. maxima* PFP complex βγ-CAT is a secretory endolysosome channel protein that has evolved to facilitate cell nutrient and water acquisition and transcellular transport while fulfilling immune defense ((24, 25, 26, 27, 31)). Considering the primary functions of βγ-CAT in *B. maxima*, there must be endogenous elements in the toad to regulate assembly and dissociation of this PFP complex, which guarantee its specific and controllable biological actions. Indeed, a previous study revealed that an endogenous paralog of βγ-CAT α-subunit BmALP1, namely BmALP3, acts as a negative regulator of βγ-CAT, which regulates protein complex dissociation and function loss relying on environmental oxygen tension (32).

The regulatory capacity of BmALP3 for BmALP1 depends on the presence of a highly conserved cysteine residue at the C-terminal part of many vertebrate af-PFPs including those from toad *B. maxima*. As a consequence, BmALP1 can be reversibly converted between active and inactive forms *via* redox reactions. BmALP3 oxidizes BmALP1 to a disulfide bond-linked homodimer that tends to form the water-soluble high molecular weight polymer. Although the BmALP1 homodimer and polymer (inactive forms) were transformed into the monomer by treatment with DTT, βγ-CAT activity was not readily restored (32). This phenomenon suggests that formation of the βγ–CAT complex requires mediation of unidentified endogenous element(s) in toad *B. maxima*. It is tempting to speculate that two steps are necessary to form the PFP complex βγ–CAT. First, a protein with a reducing capacity is essential to reduce the BmALP1 water-soluble homodimer and polymer to facilitate reduced BmALP1 production. Second, because BmALP1 monomer alone is unstable and tends to oligomerize and/or denature irreversibly, there should be a scaffold protein that binds and stabilizes the BmALP1 monomer and promotes recruitment of BmTFF3 to the scaffold-bound BmALP1 to form of the PFP complex.

Mucin-like FCGBPs are well conserved in vertebrates including humans (25, 39). The protein is widely expressed on mucosal surfaces and in external secretions with unknown physiological functions (34, 40, 41, 42). Because of the lack of anti-FCGBP antibodies, we purified the homemade anti-FCGBP antibodies for functional experiments (Fig. S6, *A*–*C*). Several lines of our experimental evidence support that FCGBP acts as a positive regulator to facilitate formation of PFP complex βγ-CAT. First, through Co-IP, pull-down assays, and BLI, all findings revealed physical interactions between FCGBP, and βγ-CAT (Fig. 1). Second, FCGBP reversed the inhibitory effect of BmALP3 on βγ-CAT, whereas Prdx6 or Trx did not reverse the inhibition of βγ-CAT by BmALP3 (Figs. 3, *A*–*C* and S3*A*). Third, FCGBP, but not Prdx6 or Trx, reduced naturally occurring BmALP1 polymer, leading to the appearance of βγ-CAT activity in the presence of BmTFF3 (Figs. 4, *A* and *B* and S4, *D*–*F*). Dye release assays of liposomes encapsulating calcein and *in vivo* experiments confirmed that FCGBP reduce polymers and assembled βγ-CAT (Fig. 4, *C* and *D*). Fourth, addition of FCGBP restored the activity of purified βγ-CAT preparations with substantial activity lose due to long-term preservation (Figs. 3, *D* and *E* and S3*B*). Finally, recombinant BmALP1 polymer was reduced and it formed the biological active βγ–CAT complex in the presence of FCGBP and BmTFF3 (Fig. S5, *A*–*C*). Taken together, it is FCGBP that possesses the capacity to reduce BmALP1 and serve as a scaffold to assemble active βγ-CAT.

**Figure 6:**
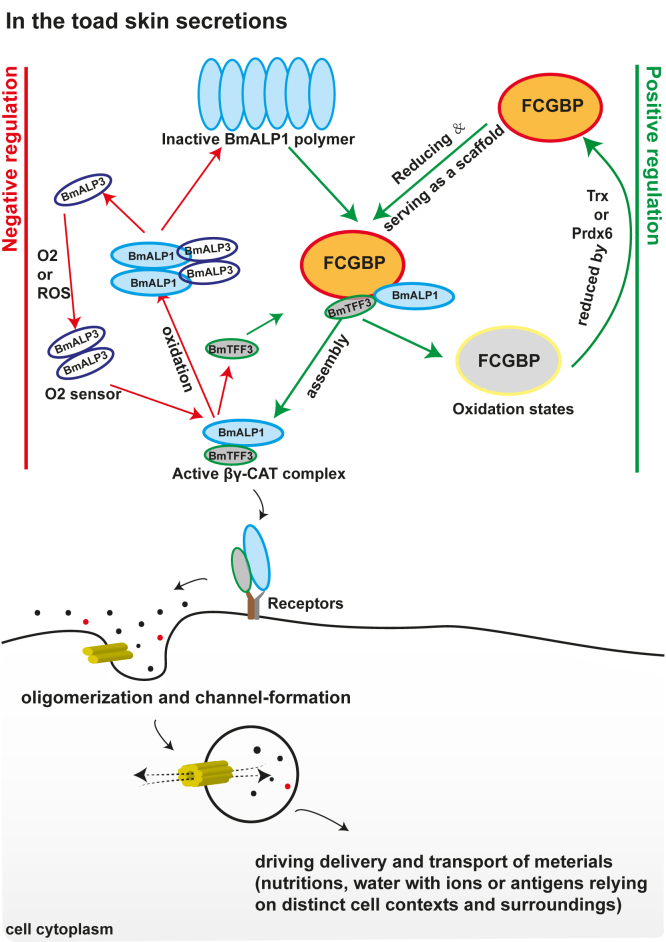
**Proposed model of FCGBP in positively regulating assembly of the PFP complex βγ-CAT.** Because of a conserved cysteine in the C terminal of af-PFPs in toad *Bombina maxima*, BmALP1 can be reversibly converted between active and inactive forms *via* redox reactions. BmALP3 senses environmental oxidative conditions (O_2_ tension and ROS levels), which converts the protein to a disulfide bond-linked homodimer. BmALP3 homodimer oxidizes BmALP1 *via* disulfide bond exchange, leading to BmALP1 polymer formation. The action negatively regulates the assembly and biological functions of the βγ–CAT complex (32). Conversely, FCGBP possesses the capacity to reduce oxidized BmALP1 and serves as a scaffold for the interaction of BmALP1 with BmTFF3 to form the βγ–CAT complex. Additionally, Prdx6 or Trx restore and enhance the effect of FCGBP on βγ-CAT assembly. ALP, aerolysin-like protein; PFP, pore-forming protein; ROS, reactive oxygen species.

FCGBP is a mucin-like protein containing repeated units composed of vWD and C8 cysteine-rich and trypsin inhibitor-like domains (Fig. 2*C*) with uniquely high-cysteine content. Similar to vWFs, there are free cystines in vWF domains of FCGBPs (43, 44, 45, 46, 47). The inactive form of BmALP1 is a disulfide bond-linked homodimer that tends to appear as the high-molecular weight polymer. Thus, free cysteines in FCGBP served as reducing elements to reduce BmALP1 polymer as observed in this study (Fig. 3). Furthermore, FCGBP provides a large scaffold to bind and stabilize the reduced BmALP1 dimer and/or monomer. The subsequent recruitment of BmTFF3 to FCGBP and its interaction with BmALP1 led to βγ–CAT complex formation, which was then released from FCGBP (Figs 1, 3 and 4, and S5). Conversely, Prdx6 and Trx were unable to directly target BmALP1 to reduce oxidized BmALP1 under our assay conditions (Fig. S3, *B* and *C* and S4, *D*–*F*), indicating the specific regulatory effect of FCGBP on BmALP1. Furthermore, the capacity of FCGBP preparations to reduce oxidized BmALP1 substantially decrease with increase in storage time, indicating oxidization of the free cysteines in the protein (Fig. 5*A*). Prdx6 or Trx restored the capacity of FCGBP to reduce polymer and mutation of the active site cysteine in Prdx6 and Trx abrogated this ability (Fig. 5, *B* and *C*). Dye release from liposomes also showed that the Trx enhanced FCGBP activity on polymers (Fig. 5*D*). These data indicated that Prdx6 and Trx reduced and released free cysteines of FCGBP (Fig. 5). These results suggest that the presence of these oxidoreductase enzymes is necessary to interact with oxidized FCGBP to restore its reducing activity. Taken together, we propose a model for reversible regulation of the PFP complex βγ-CAT by redox reactions in toad *B. maxima* dependent on environmental cues (Fig. 6).

The β-subunit (BmTFF3) of βγ-CAT is a three domain TFF identified in toad *B. maxima* (21, 23). TFFs are well-conserved from amphibians to humans (46, 48, 49). Mammalian TFF2 has been implicated in lipid intestinal absorption and anabolism (50, 51, 52). TFF peptides interact with secreted mucins (46, 49, 53, 54) and murine TFF1 interacts directly with the von Willebrand factor domains of mucins (55, 56, 57). Furthermore, human TFF1 and TFF3 form disulfide-linked heteromers with FCGBP protein with unknown biological significance (46, 57, 58, 59, 60). Consistent with the above observations, the present study determined the physical interaction of BmTFF3 with FCGBP (Fig. 1*D*), which may facilitate its recruitment and interaction with BmALP1 monomers to form the βγ–CAT complex on the FCGBP platform. FCGBP mediates the interaction of BmTFF3 with BmALP1 to form the PFP complex, which is a primary regulatory step in the βγ–CAT pathway. BmTFF3 functions in this PFP complex by acting as a chaperon that stabilizes the BmALP1 monomer and delivers BmALP1 to proper membrane targets *via* a double-receptor binding model (23, 32). Toad *B. maxima* βγ-CAT is natural example that a TFF can form a PFP complex machine working in extracellular molecule uptake and transport between a cell and the extracellular environment ((14, 24, 25, 26, 27, 31)). Considering the high conservation and functions of TFFs in vertebrates (46, 48, 50, 61, 62, 63, 64), our results provide new information on their first-hand actions and mechanisms involved in participating in novel PFP functions.

The present study revealed a newly defined a cell vesicular delivery system driven by a PFP, namely the βγ–CAT pathway, including FCGBP, Prdx6, and Trx. Together with βγ-CAT negative regulator BmALP3 (36), these proteins comprise a complicated and meticulous regulatory network of extracellular regulation of the βγ–CAT pathway (Fig. 6), highlighting the necessary and primary roles of the PFP complex in toad *B. maxima* physiology. The conserved C-terminal cysteine residue responsible for the redox regulation of BmALP1 is highly conserved in numerous vertebrate af-PFPs from fish to reptiles, and was uniformly replaced by a serine residue in diverse bird species (32), emphasizing again the important role of this site in the regulation of vertebrate af-PFPs. Accordingly, it is tempting to predict that af-PFPs with a cysteine residue at the site may be regulated by a redox mode similar to that observed in af-PFPs in toad *B. maxima*. For birds, af-PFPs phosphorylation of the serine residue might be an alternative regulatory mechanism. Our findings should serve as clues to investigate the possible existence of βγ-CAT-like cell vesicular delivery systems in diverse vertebrate species, their conservation and variation as well as biological relevance, especially those acting in cell nutrient acquisition and vesicular trafficking while fulfilling immune defense.

In conclusion, the present study elucidated that toad *B. maxima* FCGBP positively regulates assembly of the PFP complex βγ-CAT (Figure 3, Figure 4, Figure 5 and S5). In contrast to the negative regulation of βγ-CAT by its PFP paralog BmALP3 by oxidizing BmALP1, FCGBP reduces the BmALP1 polymer produced by oxidation and serves as a scaffold for assembly of the PFP complex βγ-CAT. Together with Prdx6 and Trx, these components guarantee sustainable assembly of βγ-CAT under necessary environmental conditions. Our findings define the role of FCGBP in mediating assembly of active βγ-CAT, a secretory multifunctional PFP complex machine.

## Experimental procedures

### Animals

*B. maxima* were caught in the forests of Chuxiong. Toad skin secretions were collected as described (32). Kunming mice and rabbits were purchased by Chushang Biotechnology Company. All animal procedures and experiments were approved by the Ethics Committee of Kunming Institute of Zoology, Chinese Academy of Sciences (IACUC-OE-2021-05-001).

### Sequence alignments

Full-length human FCGBP was downloaded from the UniProt database (www.uniprot.org). Full-length *B. maxima* FCGBP was generated from transcriptome and proteome databases of *B. maxima* skin from previous study (35). The amino acids sequences of FCGBP and human FCGBP were aligned by MEGX X (65). Sequence alignments were performed by Genedoc (https://genedoc.software.informer.com/; 66) and Adobe Illustrator CC.

### Immunoprecipitation/liquid chromatography/mass spectrometry

The products of IP were analyzed by LC-MS method (67). IP products were digested into peptides by 10 ng/μl trypsin at 37 °C. The peptides were separated by the EASY-nLC 1200 TM system (Thermo Fisher Scientific). The isolated peptides were analyzed by a Q Exactive HF-X mass spectrometer (Thermo Fisher Scientific). Vertical coordinates of the mass spectrum represented the relative intensity or peak intensity (AU) and the *X*-axis represented the retention time (min). The peak of each peptide in the mass spectrum was regarded as Gaussian functions, using Proteome Discoverer version 2.2 (Thermo Fisher Scientific; www.thermofisher.cn/order/catalog/product/cn/zh/OPTON-31101) software to calculate the peak area of each peptide under the Gaussian function curve. The abundance values indicated the sum of peak areas of peptides from the same protein. We averaged the abundance values of two replicates in one experiment and subtracted the average abundance values of control groups. The abundance values of differential proteins between polyclonal anti-βγ-CAT antibody and IgG control groups were obtained. These potential proteins interacted with βγ-CAT were performed by GraphPad prism (www.graphpad.com/) and Adobe illustrator CC.

### Mass spectrometry

MS performed as described (32). Briefly, protein samples were subjected to SDS-PAGE with silver or Coomassie staining. Gels were cut into pieces for destaining and enzymatic digestion into peptide fragments. For gels stained by Coomassie, gel pieces were first destained in buffer (25 mM NH_4_HCO_3_ and 50% CH_3_CN). For gels stained by silver, the gel pieces were treated with 15 mM K_3_Fe (CN)_6_ and 50 mM Na_2_S_2_O_3_. Gels were dehydrated in CH_3_CN and treated with DTT at 37 °C for 1 h. After cooling to room temperature and discarding the buffer, they were alkylated by 30 mM iodoacetamide while protected from light for 45 min. The gel pieces were washed with Milli-Q water and dried in 50 μl CH_3_CN for digestion in trypsin solution at 4 °C overnight. MS/MS data were acquired by an Auto-Flex Speed matrix-assisted laser desorption/ionization two-stage time-of-flight mass spectrometer (Bruker Daltonik GmbH).

### Molecular cloning of BmALP1 and FCGBP

The semiquantitative PCR has been described (32). Briefly, before cloning BmALP1 and FCGBP genes from different tissues, mRNAs were extracted from *B. maxima* tissues using an RNA purification kit (Promega) and reverse transcribed to cDNA using a one-strand cDNA synthesis kit (Vazyme). In accordance with the different tissue cDNAs as PCR templates and using corresponding primers, BmALP1, FCGBP, BmTFF3, and β-tubulin genes were amplified by Taq enzyme (Takara). The primer pairs were shown in Table S1.

### Recombinant vWD domain (Gly^1606^ to Val^1774^) of FCGBP protein expression and purification

The vWD domain (Gly^1606^ to Val^1774^) of FCGBP was cloned from *B. maxima* skin cDNA into the pET-28a (+) vector that was used to transform *E. coli*. Cells were grown to an A_600_ of 0.8 at 37 °C and then treated with 1 mM IPTG to induce target protein expression for 20 h at 16 °C. Harvested *E. coli* pellets were resuspended in precooled equilibration buffer (50 mM Tris–HCl and 0.5 M NaCl, pH 7.5) for ultrasonication. The ultrasonicated supernatant was applied to pre-equilibrated Ni-NTA affinity chromatography. Miscellaneous proteins were removed by washing buffer of various concentration gradients of imidazole (10, 50, and 100 mM). Samples were eluted with elution buffer (equilibration buffer containing 300 mM imidazole). The purified recombinant protein was dialyzed in PBS.

### Preparation, purification, and ELISA analysis of antibodies

Because of the lack of anti-FCGBP antibodies, we expressed and concentrated the recombinant vWD domain (Gly^1606^ to Val^1774^) of FCGBP to 2 mg/ml to immunize white rabbits (male, 2 kg). The rabbits were immunized with 1 mg protein mixed with complete Freund’s adjuvant (Sigma-Aldrich) at a 1:1 ratio. After 12 days, the rabbits were injected again with 1 mg protein mixed with incomplete Freund’s adjuvant (Sigma-Aldrich) at a 1:1 ratio. Rabbits were boosted every 7 days until anti-FCGBP antibodies had high antibody specificities and titers. Blood from the rabbit heart was centrifuged (2100*g*, 20 min) at 4 °C to collect antiserum. Samples on SDS-PAGE were *B. maxima* skin secretion. The rabbit anti-FCGBP antibodies were purified from anti-serum by antigen-immunoaffinity chromatography by incubation with protein A resin (TransGen Biotech) pre-equilibrated in PBS and eluted with 0.1 M Glycine (pH 3.0). The anti-FCGBP antibodies were dialyzed in PBS at 1 mg/ml.

The titers were measured by an indirect sandwich ELISA. The vWD domain (Gly^1606^ to Val^1774^) of FCGBP and *B. maxima* skin secretion were diluted in ELISA coating buffer (Solarbio) to a final concentration of 10 μg/ml to coat 96-well plates (100 μl/well) at 4 °C overnight. The plates were washed five times with 200 μl PBST (PBS, 1% Tween-20) for 2 min and blocked with 5% bovine serum albumin (BSA) for 2 h at 37 °C. After washing by PBST, the serial dilution of anti-FCGBP antibodies with 0.1% BSA was added into each well, followed by incubation at 37 °C for 2 h. After washing at least five times with PBST, 100 μl horseradish peroxidase-conjugated goat anti-rabbit IgG (Proteintech) at a 5000-fold dilution in 0.1% BSA/PBST was added into each well and incubated at 37 °C for 1 h. After washing five times with PBST, 20 μl component A buffer of a TMB Two-Component Substrate solution kit (Solarbio) was applied for 20 min and then 80 μl component B buffer was added to stop the reaction. The plates were analyzed at 450 nm using an infinite 200 PRO microplate reader (Tecan). The purify of anti-FCGBP polyclonal antibodies was analyzed by 10% SDS-PAGE. The purification and identification of anti-FCGBP antibodies were shown in Fig. S6.

Rabbit polyclonal antibodies against βγ-CAT and BmTFF3 were produced as described (32).

### Expression and purification of recombinant BmALP1 protein

BmALP1 gene was ligated into a pPIC9K plasmid by Tsingke Biotech and expressed with a His6-Tag through *Pichia Pastoris* GS115. Single yeast colonies of transformants on the plate of yeast extract peptone dextrose medium were inoculated in BMGY medium and grown to an OD_600_ of 3.0 in a shaking incubator (250 rpm, 30 °C). After centrifuging at 3000*g* for 10 min at 4 °C, the yeast was resuspended in BMMY broth and induced for 96 h in the shaking incubator (250 rpm, 30 °C) in a final concentration of 1% methanol. After centrifugation (10,000*g*, 10 min) at 4 °C and adjusting the pH of the supernatant to 7.5 using 1 M Tris–HCl, the supernatant was loaded onto a pre-equilibrated Ni-NTA column (GE healthcare) with buffer (50 mM Tris–HCl and 0.5 M NaCl, pH 7.5). After the column was washed with various concentration of equilibration buffer containing imidazole (10, 50, and 100 mM), it was eluted by equilibration buffer-containing 300 mM imidazole. The purified recombinant protein was dialyzed in PBS for experiments. Identification of the protein was performed by Western blotting and hemolysis assays.

BMGY medium (100 ml): 1 g yeast extract (OXOID), 2 g peptone (Solarbio), 10 ml of 13.4% yeast nitrogen base (Solarbio), 10 ml of 10% glycerol, 10 ml of 1 M K_2_HPO_4_/KH_2_PO_4_, pH 6.0, and 0.2 ml of 0.02% biotin.

BMMY broth (100 ml): 1 g yeast extract (OXOID), 2 g peptone (Solarbio), 10 ml of 13.4% yeast nitrogen base (Solarbio), 10 ml of 10% methanol, 10 ml of 1 M K_2_HPO_4_/KH_2_PO_4_, pH 6.0, and 0.2 ml of 0.02% biotin.

PBS (pH 7.4, 1000 ml): 8 g NaCl, 0.2 g KCl, 1.44 g Na_2_HPO_4_, and 0.24 g KH_2_PO_4._

### Sepharose-4B antibody affinity chromatography

Sepharose-4B coupled with antibody was prepared by a described method (36). Briefly, swelled and CNBr-activated Sepharose-4B gel (GE healthcare) was incubated with antibodies in coupling buffer (0.1 M NaCO_3_ and 0.5 M NaCl, pH 8.0) overnight at 4 °C. Washing uncoupling antibodies and blocking nonspecific groups on the coupling antibody column were carried out in accordance with the manufacturer’s protocol.

Prepared anti-βγ-CAT and anti-FCGBP antibody-Sepharose-4B were packed into columns (1.56 × 8.3 cm) in accordance with the manufacturer’s protocol. Purified samples from anion exchanges were loaded onto the antibody-affinity column. After washing the column by equilibrated buffer and washing buffer at two concentrations of NaCl (100 and 200 mM), samples were eluted with elution buffer (50 mM Tris and 1 M NaCl, pH 8.0). The elution fractions were dialyzed and concentrated in PBS for experiments.

### Purification of FCGBP

*B. maxima* skin secretions were collected as described (32). The powder of skin secretions was dissolved in PBS at 40 mg/ml and loaded onto a gel filtration Sephadex G-100 column (4.6 × 100 cm) with 0.15 ml/min flow. Fractions were collected every 15 min. The purify of fractions was evaluated by SDS-PAGE and identification of FCGBP was performed by Western blotting. Peak I of the Sephadex G-100 column was concentrated and dialyzed in the equilibration buffer (50 mM Tris–HCl, pH 8.2) for the following preparation of anion exchange. The sample was loaded onto a pre-equilibrated Resource Q column (GE healthcare) and eluted by a linear gradient of NaCl to collect 2 ml per tube. Peak III of resource Q was loaded onto a pre-equilibrated anti-FCGBP antibody-affinity column, with various gradients of NaCl (0, 100, and 200 mM) used to extensively wash. The column was eluted by elution buffer (50 mM Tris and 1 M NaCl, pH 8.0). Elution fractions were dialyzed and concentrated in PBS for follow experiments. The purity and identification of FCGBP were performed by SDS-PAGE with silver staining and Western blotting, respectively.

### Purification of βγ-CAT and BmTFF3

*B. maxima* skin secretions were collected as described (32). Purification and analysis of BmTFF3 and βγ-CAT were carried out in accordance with previous studies (21, 23).

### Recombinant Prdx6, Prdx6 mutant, Trx, and Trx mutant

Genes encoding Prdx6 and Trx were cloned from toad *B. maxima* skin cDNA into the pET-28a (+) vector with an N-terminal His_6_-Tag. rePrdx6 ^(C46A, C90A)^ and reTrx ^(C32A, C35A)^ mutants were cloned by point-mutation PCR into the pET-28a (+) expression vector with an N-terminal His_6_-Tag. Competent *E. coli* strain Rosetta (DE3) (TsingKe) was transformed with the recombinant plasmids.

Transformed colonies were grown to an A_600_ of 0.8 in a shaking incubator (200 rpm, 37 °C) and then treated with 2 mM IPTG to induce target protein expression for 20 h in a shaking incubator (200 rpm, 16 °C). *E. coli* pellets was resuspended in precooled equilibration buffer (50 mM Tris–HCl and 0.5 M NaCl, pH 7.5) for ultrasonication. Supernatants were collected by centrifuging at 16,000*g* for 40 min. The supernatant was loaded onto a pre-equilibrated Ni-NTA affinity chromatography column (GE healthcare). Various concentrations of imidazole (equilibration buffer containing 10, 50, and 80 mM imidazole) were applied to wash extensively. Columns were eluted by equilibration buffer containing 300 mM imidazole. Purified recombinant proteins were dialyzed and concentrated in PBS for hemolysis assays.

### Western blotting

Samples for Western blotting were pretreated as follows: in Figure 3, BmALP3 prior to reacting with βγ-CAT; in Figure 4, BmALP1 polymers prior to mixing with BmTFF3; and reBmALP1 polymers prior to reacting with BmTFF3. The mixture was respectively incubated with FCGBP for 10 min at room temperature. Samples were mixed with a loading buffer to load on SDS-PAGE gels. Pretreated protein samples were run on SDS-PAGE gels under nonreducing conditions and transferred to polyvinylidene fluoride (PVDF) membranes (Merck). PVDF membranes were blocked with 5% skimmed milk for 2 h at room temperature. After washing three times with the tris buffered saline with Tween-20 (TBST) buffer, PVDF membranes were incubated with various concentrations of rabbit antibodies overnight at 4 °C, including rabbit IgG, anti-βγ-CAT, anti-FCGBP, and anti-BmTFF3 antibodies. After washing three times in TBST buffer, PVDF membranes were incubated with horseradish peroxidase-conjugated goat-anti-rabbit IgG (H + L) for 2 h at room temperature. After washing three times in TBST buffer, PVDFs were subjected to a Tannon machine with a High-Sig Enhanced Chemiluminescence Western blotting substrate kit (Tannon). The switched quantities of BmALP1 monomer bands were calculated by ImageJ (https://imagej.net/) as described (32).

TBST (pH 7.4, 1000 ml): 8 g NaCl, 0.2 g KCl, 3 g Tris-base, and 1% Tween-20.

### Co-IP assays

Co-IP was conducted using a Co-IP kit (Proteintech). Briefly, *B. maxima* skin secretions (3 mg/ml, 300 μl) were incubated with 4 mg antibodies (anti-βγ-CAT, anti-FCGBP, anti-BmTFF3) or rabbit IgG overnight at 4 °C on a rotating mixer (Crystal & Technology). The mixture was applied to the same amount of prepared protein A/G beads slurry for 3 h at 20 °C on the rotating mixer. Suspensions with protein A/G beads were placed on spin columns, washed ten times with washing buffer, and placed spin columns in a 1.5-ml eppendorf tube to collect the elution product. Beads were incubated twice with 40 μl elution buffer at room temperature for 10 min and then centrifuged at 10,000×*g* at 4 °C for 2 min. The elution was mixed with 10 μl Alkali neutralization buffer.

### Bio-layer interferometry binding kinetics assays of FCGBP and βγ-CAT

The molecular interaction of purified FCGBP with βγ-CAT was identified by the BLI method on an Octet system (Sartorius). The method was performed in accordance with studies (68, 69) with some modifications. An amine Reactive second generation (AR2G) biosensor was run at baseline in PBS for 300 s, activated in 100 μl EDC/NHS (20 mM 1-ethyl-3-(3-dimethylaminopropyl) carbodiimide and 10 mM N-hydroxysuccinimide) for 300 s, loaded in 100 μl FCGBP (0.01 M sodium acetate, pH 4.0) at 10 μg/ml for 600 s, quenched in 1 M ethanolamine for 300 s, and run at baseline again for 120 s. After preliminary experiments of FCGBP (10 μg/ml) and βγ-CAT (1 μM) to calculate binding kinetic constants, a 2-fold dilution of various concentrations of βγ-CAT (1000, 500, 250, 125, 62.5, 31.25, and 0 nM) dissolved in PBS was run at association for 300 s and PBS was run at dissociation for 180 s. Each were added at 100 μl to a black polypropylene 96-well microplate (Greiner Bio-one). Acquired data were loaded by Octet Data Analysis Studio software (Sartorius; https://www.sartorius.com/en/products/protein-analysis/octet-bli-detection/octet-systems-software), processing the data by specifying steps: selecting a reference sensor and reference wells for reference subtraction, *y*-axis alignment to the baseline, inter-step correction of Dissociation, and Savitzky–Golay filtering. The processed data were analyzed in the Analysis window by specifying steps: clicking the analyzing Association and Dissociation steps, fitting the group by a global fit and a 1:1 binding model, clicking R_max_ unlinked by the sensor, and using the window of interest to select step times, clicking the fit curves button to calculate the equilibrium dissociation constant (*K*_D_), *K*_D_ error, full X^2^, and full R^2^. The analyzed data were exported to generate a detailed report in Microsoft Excel. *K*_D_ indicated and measured how tightly the ligand binds to its analyte. A smaller *K*_D_ indicated greater affinity of βγ-CAT to FCGBP. Errors, X^2^, and R^2^ were used to evaluate the quality of the fit and reliability of calculated binding and affinity constants. Error values were acceptable when they were within one order of magnitude of the rate constant values. R^2^ values indicated how good the fit and the experimental data correlate, which were considered a good fit when the value was above 0.95. X^2^ value was the sum of the squared deviations and indicated the error between the experimental data and the fitting line. A smaller X^2^ indicated a better fit and commonly should be below three.

Association and dissociation data of βγ-CAT were exported alone from 1620 to 2099.8 s and autozeroed from 0 s to 479.8 s. Images were constructed by GraphPad Prism, including curves of βγ-CAT with five concentrations (1000, 500, 250, 125, and 62.5 nM) and excluding curves of the reference wells (0 nM) and the lowest concentration (31.25 nM) of βγ-CAT.

### Pull-down assays

Anti-FCGBP antibody-Sepharose-4B and rabbit IgG-Sepharose-4B packed in spin columns were incubated with *B. maxima* skin secretions overnight at 4 °C in PBS. After washing with 10-column volumes of PBS, the spin columns were placed in 1.5-ml EP tubes and eluted three times by elution buffer (PBS containing 1 M NaCl).

### Hemolysis assays

The method of hemolysis assays was referred to the previous research (32). For the pretreated samples in hemolytic assays of Figure 3, *A* and *B*, βγ-CAT was preincubated with BmALP3 or BmTFF3. In Figure 3*B*, these pretreated samples were then incubated with various concentrations of FCGBP (0, 5, 10, and 30 nM) for 10 min at room temperature. In Figure 3*E*, the samples (240-day-stored βγ-CAT at 4 °C and fresh FCGBP at various concentrations) were mixed for 10 min at room temperature. For pretreated samples in hemolytic assays of Figure 5, polymers were preincubated with BmTFF3 or FCGBP and then incubated with reprdx6, reTrx, or their mutants for 10 min at room temperature. The mixtures were incubated with human RBCs (6 × 10^7^ cells/ml) in 200 μl PBS at 37 °C for 30 min. After the reaction solutions were centrifuged at 500*g* for 5 min to collect the supernatants, absorption at 540 nm was measured. The hemolysis % of Max (%) was calculated by the equation:

$$\text{The}\;\text{hemolysis}\;\%\;\text{of}\;\text{Max}\left(\%\right)=\left(A_{\text{sample}}-A_{\text{negative}\;\text{control}}\right)/A_{\text{positive}\;\text{control}}\times 100\text{.}$$

where A _sample_ is absorbance at 540 nm, and A _positive control_ is the 100% maximum absorbance value when 0.1% Triton X-100 were incubated with human RBCs (6 × 10^7^ cells/ml), and A _negative control_ is absorbance values of human RBCs (6 × 10^7^ cells/ml) treated with PBS.

### Preparation of calcein-encapsulated liposomes

Liposomes encapsulating calcein at a selfquenching concentration (120 mM) were prepared to detect calcein leakage and identify βγ-CAT formation. Liposomes were composed of phosphatidyl choline (100% PC). The method was mainly referred as references (70, 71) with some modifications. PC (Picasso) was dissolved in a methanol/chloroform mixture at a 1:9 ratio with a final concentration of 35 mg/ml and protected from light in distillation flasks. The dissolved PC was dried in a rotary evaporator (Heidolph) to form a lipid film. Dry lipid films were hydrated in MES (120 mM calcein, 50 mM MES, and 150 mM NaCl, pH 7.2) at a final concentration up to 10 mg/ml. The lipid mixture was swirled for 2 h (180 rpm, 37 °C) and then subjected to five cycles of freezing and thawing in liquid nitrogen and a 50 °C water bath. To make the liposome particle size uniform, 10 mg/ml hydrated lipid solution was extruded through two stacked polycarbonate membrane filters with pore sizes of 100 nm (Whatman) for ten to thirty times on a Mini-Extruder (Avanti Polar Lipids). Nonencapsulated calcein was removed out through five to eight cycles of washing with MES buffer without calcein in 100 kDa filtration tubes (Merck).

The liposomes were made out to store at 4 °C and used for dye-release assays within 48 h. Leakage of calcein from the liposomes was detected by the fluorescence intensity at 518 nm emission. Because abundant liposomes were needed to perform dye release assays, the polymer (50 nM), BmTFF3 (200 nM), and various concentrations of FCGBP (25, 50, and 150 nM) were applied to 10 μg liposomes in 100 μl MES buffer (50 mM MES and 150 mM NaCl, pH 7.2). The fluorescence intensity of Max (%) was calculated by the equation:

$$\text{The}\;\text{fluorescence}\;\text{intensity}\;\text{of}\;\text{Max}\;\left(\%\right)=I_{\text{sample}}/I_{\text{maximum}}\times 100\text{.}$$

where I _sample_ is the fluorescence intensity at 2 min, and I _maximum_ is the total fluorescence intensity after addition of Triton X-100 (90 μl of 0.1% Triton X-100 is added to each 100 μl sample).

Measurements were obtained on a multimode microplate reader, Infinite M 1000 PRO instrument (Tecan, PerkinElmer) (λ _excitation_ = 495 nm, λ _emission_ = 518 nm).

### *In vivo* mouse peritoneum experiments

Polymer (24.1 mg/kg), BmTFF3 (27.5 μg/kg), and FCGBP (75 μg/kg) were injected in the mouse peritoneum (35 ± 5 g) for 4 h with 0.9% NaCl as the negative control and βγ-CAT (28.5 μg/kg) as the positive control. Mice were injected with 5 × 10^7^ colony-forming units/ml *E. coli* (ATCC 25922) in accordance with a previous study (30) with some modification. After 11 h of bacterial infection, mice were sacrificed and injected with 1 ml of 0.9% NaCl to harvest peritoneal lavage fluids for bacterium counting.

Harvested peritoneal lavage fluids were serially diluted by using 100-fold dilution technique. 1 × 10^−8^ dilution of peritoneal lavage fluids (100 μl) were plated out in LB plates at 37 °C for 12 h. Distinct colonies were expressed as log colony-forming units/ml.

### Statistics

Values are the mean ± SD. The data were at least repeated twice and performed by represent experiments. Data analysis was conducted by GraphPad Prism software. Statistical differences of two groups comparisons were analyzed by the unpaired *t* test. Multiple comparisons of more groups were conducted by ANOVA analysis. Differences with *p* value < 0.05 were statistically significant.

## Data availability

The raw MS files of LC/MS and the BLI data are available through Zenodo accession No. 7716634 and 7812078. All remaining data are contained within the article.

## Supporting information

This article contains supporting information.

## Conflict of interest

The authors declare that they have no conflicts of interest with the contents of this article.
