# Supplementary material for: IgG Fc-binding protein positively regulates the assembly of pore-forming protein complex βγ-CAT evolved to drive cell vesicular delivery and transport
Source: J Biol Chem. 2023 Apr 15;299(6):104717. doi: 10.1016/j.jbc.2023.104717 (PMC10206775; doi:10.1016/j.jbc.2023.104717)
Supplement: Supporting Figures S1–S6 and Table S1 [file mmc1.pdf]

Supporting Information

IgG Fc-binding protein positively regulates the assembly of pore-forming protein complex βγ-CAT evolved to drive cell vesicular delivery and transport

Xianling Bian[1,2], Ziru Si[1,2], Qiquan Wang[2,4], Lingzhen Liu[2], Zhihong Shi[2], Changlin Tian[1]*, Wenhui Lee[2]*, and Yun Zhang[2,3]*

[1] Hefei National Laboratory for Physical Sciences at Microscale, School of Life Sciences, Division of Life Sciences and Medicine, University of Science and Technology of China, Hefei, Anhui 230027, China.

[2] Key Laboratory of Animal Models and Human Disease Mechanisms of the Chinese Academy of Sciences/Engineering Laboratory of Peptides of the Chinese Academy of Sciences, Institute of Zoology, the Chinese Academy of Sciences, Kunming, Yunnan 650201, China.

[3] Center for Excellence in Animal Evolution and Genetics, Chinese Academy of Sciences, Kunming, Yunnan 650201, China.

[4] Human Aging Research Institute (HARI) and School of Life Sciences, Nanchang University, Nanchang, Jiangxi 330031, China.

* Author to whom correspondence should be addressed:
Dr. Yun Zhang and Dr. Wenhui Lee
Kunming Institute of Zoology, the Chinese Academy of Sciences
No.17 Longxin Road, Kunming, Yunnan 650201, China
Tel: +86-871-65194279; Fax: +86-871-65191823
E-mail: **zhangy@mail.kiz.ac.cn; leewh@mail.kiz.ac.cn;**
Dr. Changlin Tian
Hefei National Laboratory for Physical Sciences at Microscale, School of Life Sciences, Division of Life Sciences and Medicine, University of Science and Technology of China, Hefei, Anhui 230027, China
E-mail: **cltian@ustc.edu.cn；**

Figure S1 provides the results of the use in this study.

Figure S2 provides the results related to figure 2 including the purified process and sequence alignment of FCGBP.

Figure S3 provides the results related to figure 3 including the controls of hemolysis assays and prdx6 or Trx working no effect on the inhibition of BmALP3 on βγ-CAT.

Figure S4 provides the results related to figure 4 including the purification of natural polymers, the controls of hemolysis assays, prdx6 or Trx working no effect on the assembly of βγ-CAT.

Figure S5 provides the results that FCGBP assembles the active βγ-CAT complex by recombinant BmALP1 and BmTFF3.

Figure S6 provides the identified results of anti-FCGBP antibodies used in this study.

Table S1 provides the results of sequences of primers used in this study.

A

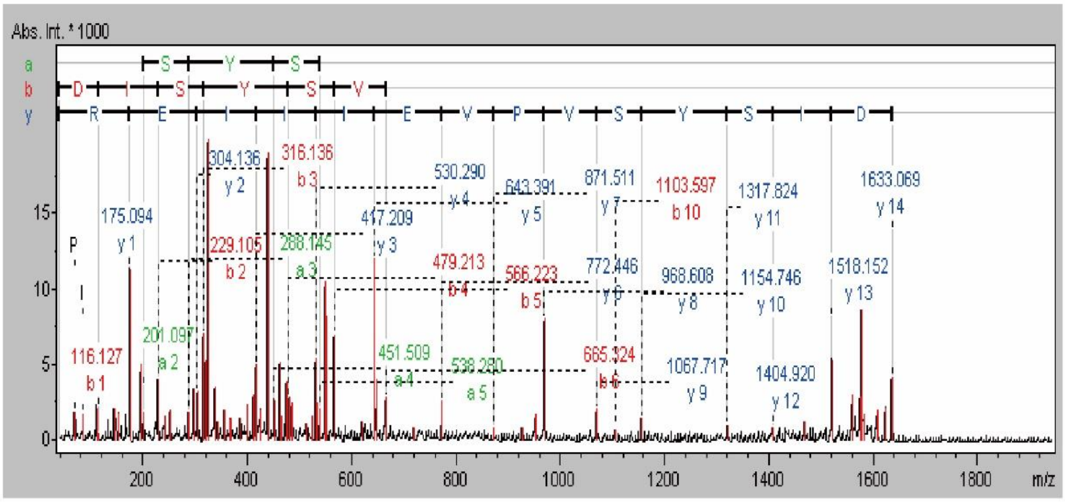

Figure S1. Related to Fig. 1. **(A)** The MS data of a BmALP1 peptide (DISYSVPVEIIIER) was determined by mass spectrum.

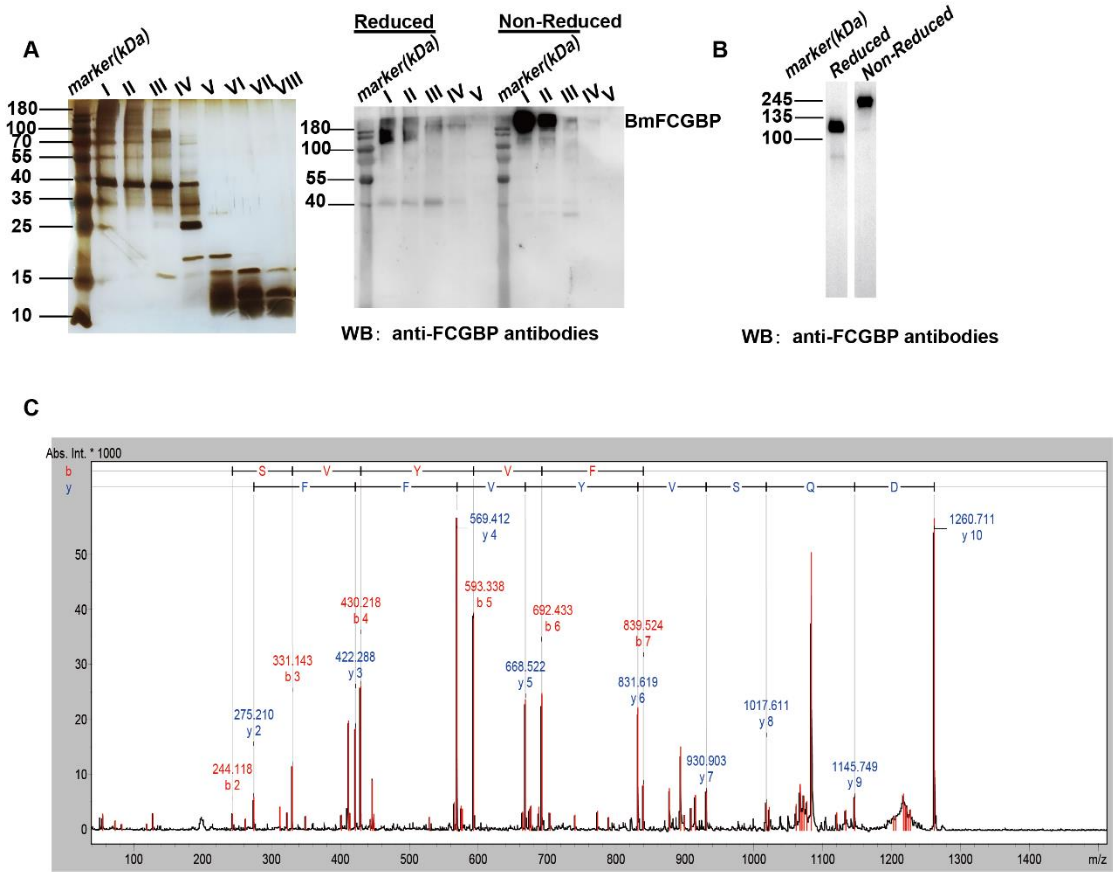
A
marker(kDa)
I II III IV V VI VII VIII
180
100
70
55
40
35
25
15
10
Reduced
Non-Reduced
marker(kDa)
I II III IV V
marker(kDa)
I II III IV V
180
100
55
40
BmFCGBP
WB: anti-FCGBP antibodies
B
marker(kDa)
Reduced
Non-Reduced
245
135
100
WB: anti-FCGBP antibodies
C
Abs. Int. * 1000
b
y
S V Y V F
F F V Y V S Q D
569.412 y 4
1260.711 y 10
593.338 b 5
430.218 b 4
692.433 b 6
839.524 b 7
331.143 b 3
422.288 y 3
668.522 y 5
831.619 y 6
275.210 y 2
1017.611 y 8
244.118 b 2
930.903 y 7
1145.749 y 9
m/z

D

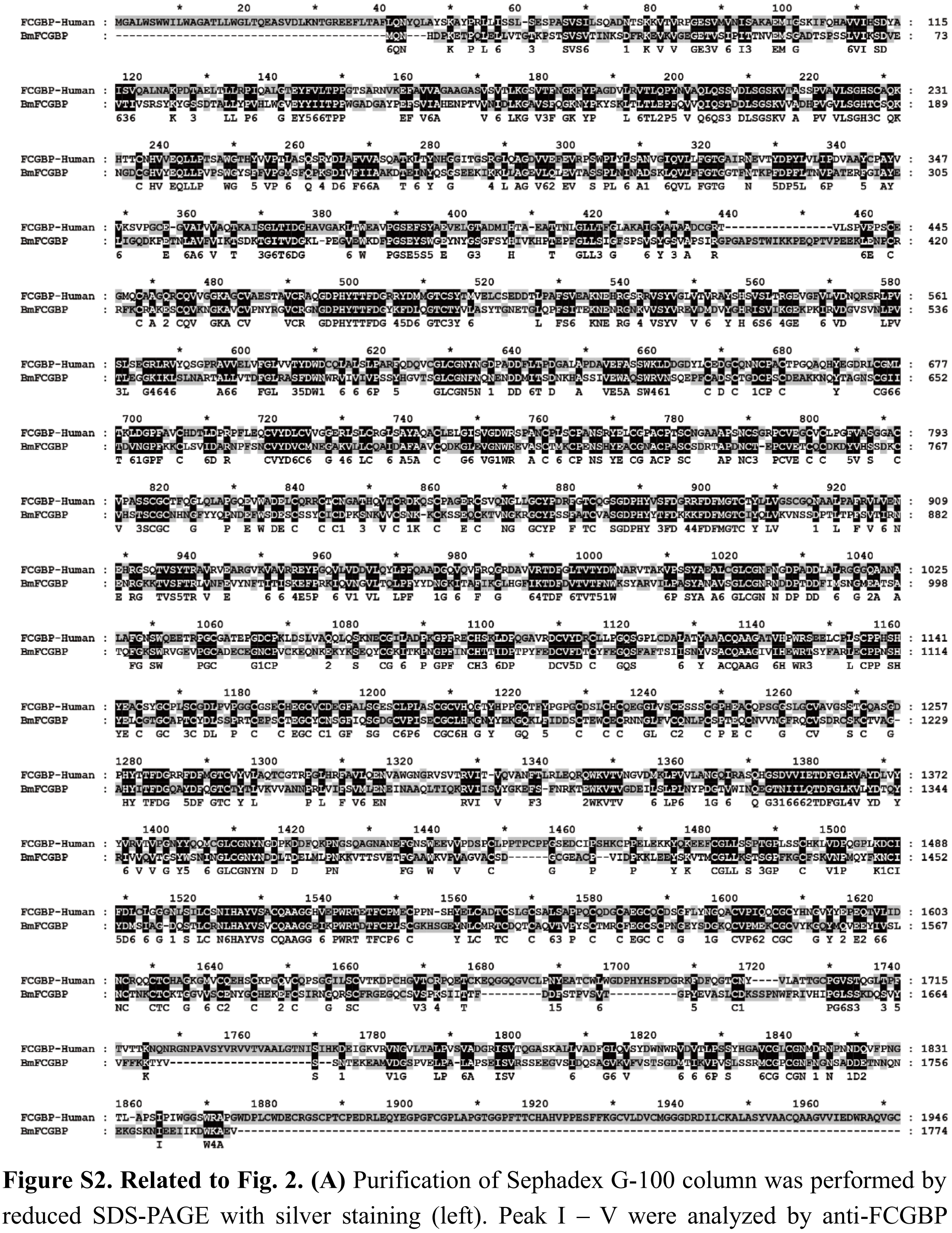

Figure S2. Related to Fig. 2. (A) Purification of Sephadex G-100 column was performed by reduced SDS-PAGE with silver staining (left). Peak I – V were analyzed by anti-FCGBP antibody of western blotting on reduced and non-reduced SDS-PAGE (right). **(B)** Related to Fig. 2C. FCGBP was performed by western blotting on reduced and non-reduced SDS-PAGE. **(C)** The peptide of FCGBP (DQSVYVFF) was identified by MALDI-TOF/TOF. **(D)** Sequence alignments on the whole sequences of FCGBP and human FCGBP (uniport ID: Q9Y6R7).

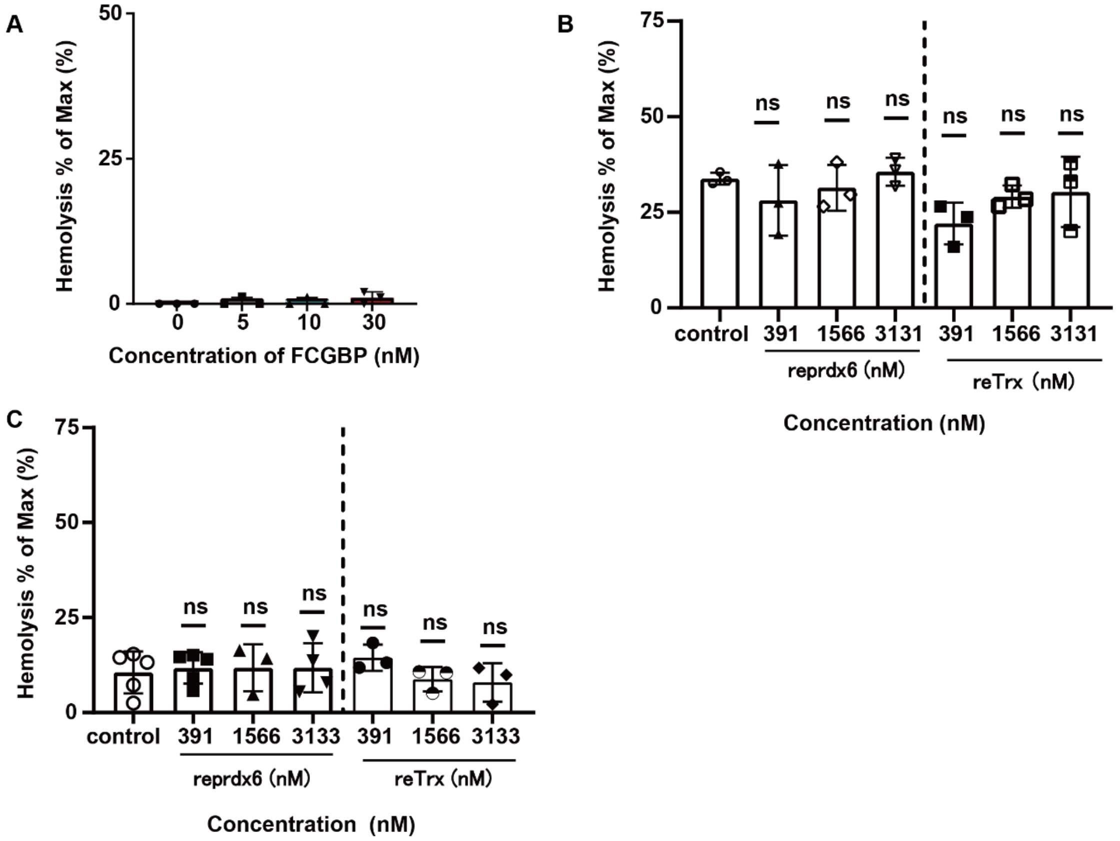

Figure S3. Related to Fig. 3. (A) Hemolysis assays on various concentrations of FCGBP incubated with human RBCs. **(B)** Various concentrations of rePrx6 or reTrx were incubated with BmALP3 (6 μM) and βγ-CAT (5 nM) and then incubated with RBCs for hemolysis assays. **(C)** 240-day-stored βγ-CAT (5 nM) was mixed with fresh rePrdx6 or reTrx at various concentrations and then was assessed by hemolysis of RBCs. All data significance represents the mean ± SD of triplicate samples. ns $p > 0.05$, * $p < 0.05$, ** $p < 0.01$, *** $p < 0.001$, and **** $p < 0.0001$ by One-Way ANOVA test.

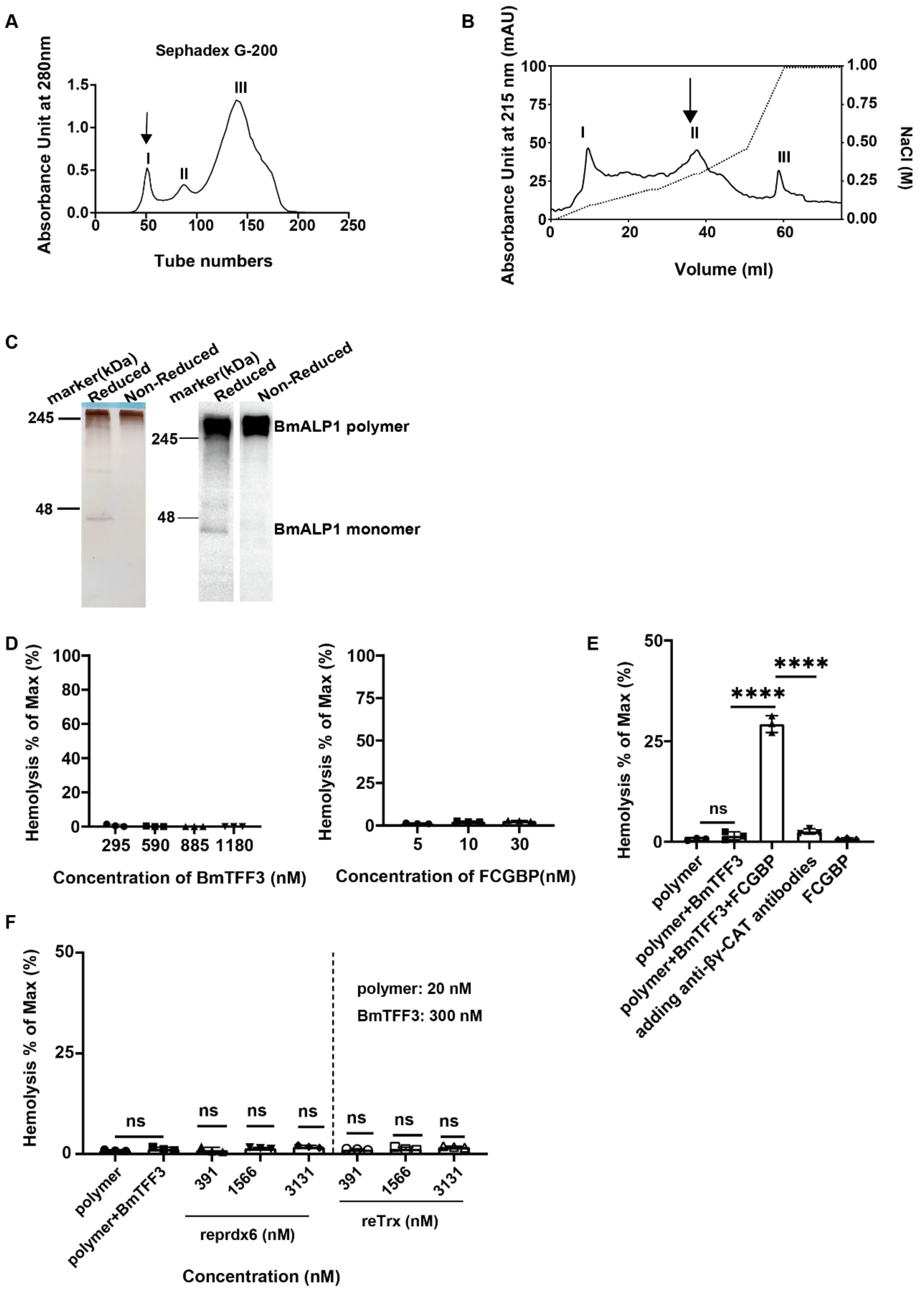

Figure S4. Related to Fig. 4. (A) Purification of natural BmALP1 polymers on Sephadex G-200 column. The arrow indicates natural polymers were found in the fraction of Peak I. **(B)** Purification of peak I containing the natural polymer on a Resource Q anion exchange column, natural polymers were arrowed on the peak II. **(C)** Natural polymers from antibody affinity chromatography of anti-βγ-CAT were performed on SDS-PAGE with silver stain (left), and analyzed by western blots (right). **(D)** Hemolysis assays on BmTFF3, FCGBP serving as

controls of Fig. 4A. **(E)** Natural polymers (20 nM), BmTFF3 (300 nM), and FCGBP (10 nM) were analyzed by hemolysis assays. The hemolysis of the mixture (polymers, BmTFF3, and FCGBP) was blocked by anti-βγ-CAT antibodies. **(F)** Various concentrations of rePrx6 or reTrx were incubated with the mixture of natural polymers and BmTFF3 and then were analyzed by the hemolysis of RBCs. All data significance represents the mean ± SD of triplicate samples. ns $p > 0.05$, * $p < 0.05$, ** $p < 0.01$, *** $p < 0.001$, and **** $p < 0.0001$ by Ordinary one-way ANOVA test.

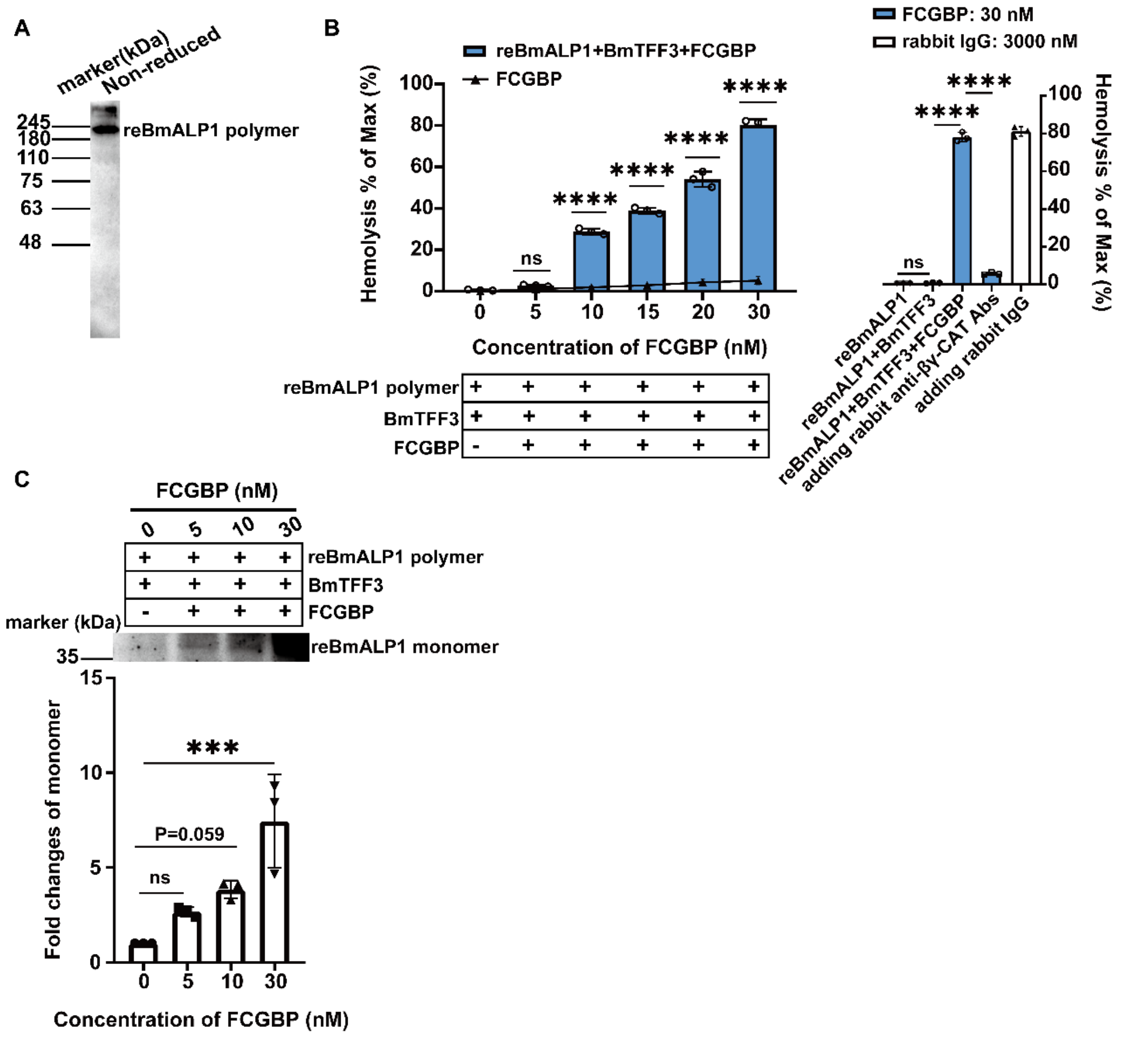

Figure S5 FCGBP assembles the active βγ-CAT complex by recombinant BmALP1 and BmTFF3. (A) Western blots of the recombinant alpha subunit (BmALP1) polymers of βγ-CAT using the anti-βγ-CAT antibody. SDS-PAGE was carried out under non-reducing conditions. **(B)** Hemolysis of human RBCs were induced by mixtures containing reBmALP1 polymers (240 nM), BmTFF3 (2.4 μM), and FCGBP at different concentrations. The black curve as the control showed hemolysis of RBCs were induced by various concentrations of FCGBP (Left). Hemolysis of RBCs were induced by mixtures of FCGBP (30 nM), reBmALP1 polymers, and BmTFF3, with anti-βγ-CAT antibodies (3 μM) or rabbit IgG as the control (right). **(C)** Changes of reBmALP1 monomers band were showed in the presence of FCGBP and BmTFF3 determined by western blots (upper). Semi-quantified changes in the amount of reBmALP1 monomers were measured by ImageJ (bottom). Reaction mixtures contained reBmALP1 polymers (200 nM), BmTFF3 (3 μM) and FCGBP. All data significance represents the mean ±

SD of triplicate samples. * $p < 0.05$, ** $p < 0.01$, *** $p < 0.001$, and **** $p < 0.0001$ by Ordinary One-Way ANOVA and Two-way ANOVA test.

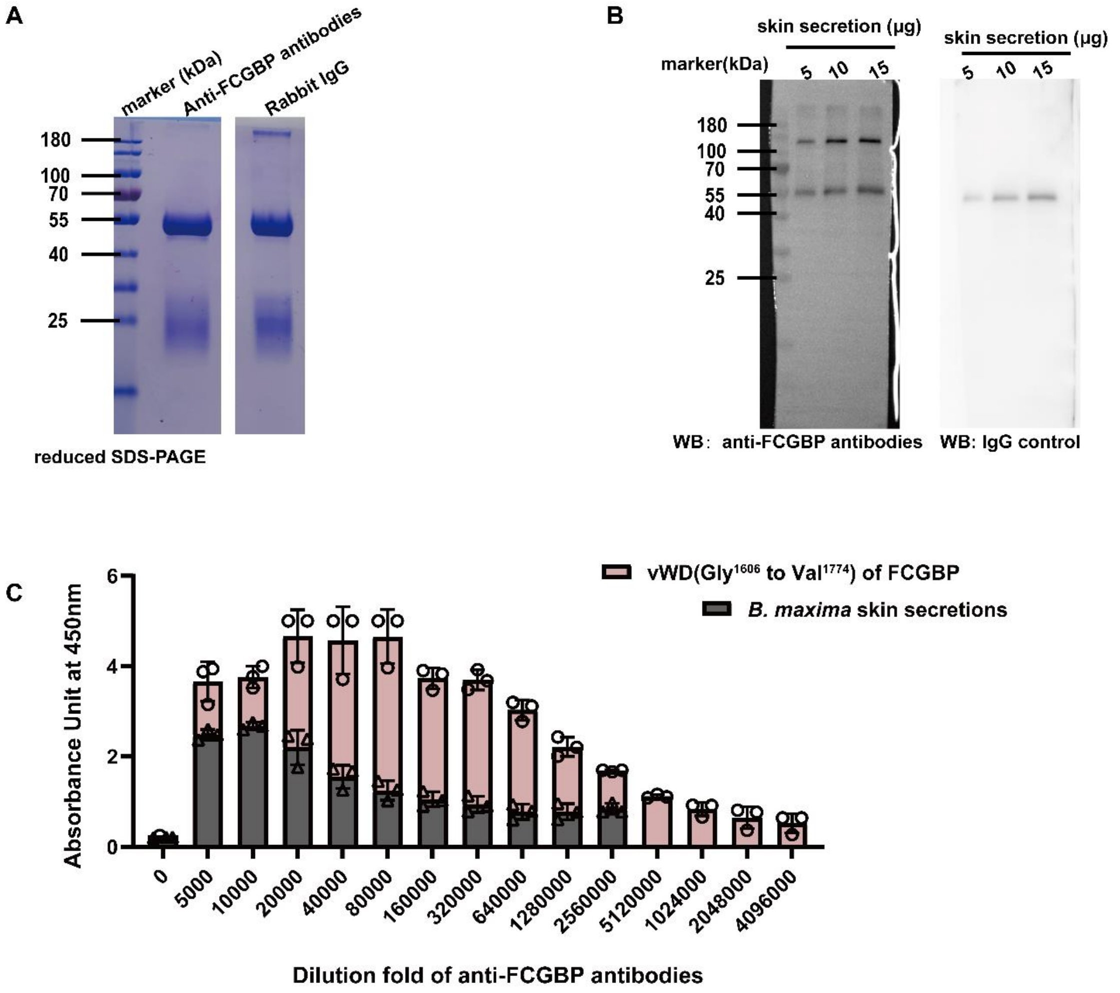

Figure S6. Preparation and purification of anti-FCGBP antibodies.

(A) The purify of anti-FCGBP antibody was identified by reduced SDS-PAGE, with rabbit IgG (1 mg/ml) as the control. **(B)** The anti-FCGBP antibodies were identified by western blots through *B. maxima* skin secretions were subjected on SDS-PAGE and antibodies were diluted by 10,000 folds. **(C)** The purified anti-FCGBP antibodies (1 mg/ml) were tested by indirect ELISA method to assess the antibody titer. Antibody titer respected a measure of the minimum concentration required for an antibody to recognize a particular epitope.

Table S1 Primers

Primers usages	Primer names	Sequences
**Co-IP**	946Alp1check_F	GATGAGCCCTCATTTGAAGGTCTGAGCTTAGAACTCAC
	946Alp1check_R	TACCATCTCTGCATATAAGCTCAGCATATTCTGTCTTC
	32aTFF3_BHF	GCTGATATCGGATCCATGGCAAGCAAGATGTTCTTC
	32aTFF3_BHR	AGTGCGGCCGCAAGCTTTTATACTTGAGGTCTAAAG
	p28VVCT_R	TGGTGGTGCTCGAGTCCGAAGCAACTGC
	p28VVCT_F	CGAATTCGAGCTCCTACACAAAGGGAATTATTATGAAAAGGG
	tublin26098_F	ATGAGGGAAATCGTGCATCTTCAAG
	tublin26098_R	ACAGGCAGCCATCATATTCTTTGCA
**rePrdx6 recombinant and mutants**	p28prx6_F	TCCGAATTCGAGCTCATGCCTGGCATTCTTCTC
	p28prx6_R	GTGGTGGTGCTCGAGTTAGTTTGGCTGTTCGG
	p28prx6C46A_F	GATTATACCCCTGTCGCGACAACAGAACTGGGTC
	p28prx6C46A_R	GACCCAGTTCTGTTGTCGCGACAGGGGTATAATC
	p28prx6C90A_R	GTTTCTGTGGGTTCTTCCGCGTTGTATGAATTTATGTC
	p28prx6C90A_F	GACATAAATTCATACAACGCGGAAGAACCCACAGAAAC
**reTrx recombinant and mutants**	28a_thx6725_F	CCGAATTCGAGCTCATGGTCAAAAAAATCGAAAATTTGGAGG
	28a_thx6725_R	GTGGTGGTGCTCGAGTTTTAACTCTTGAATCTTTTTCTCCAG
	p28ThxC32C35A-R	GAGCAATTCTTTTCGCTGGCCCCGCCCAAACTG
	p28ThxC3235A-F	CAGTTTGGGCGGGGCCAGCGAAAAGAATTGCTC
**The vWD domain (Gly^{1606} to Val^{1774}) of FCGBP recombinant**	p28a_VWFlast_F	ATCCGAATTCGAGCTCGGCGAAGGTCAATGTAG
	p28a_VWFlast_R	GGTGGTGGTGCTCGAGTTAAACTTCAGCTTTCCAGT
